# Supplementary material for: Regulation of hippocampal excitatory synapses by the Zdhhc5 palmitoyl acyltransferase
Source: J Cell Sci. 2021 May 11;134(9):jcs254276. doi: 10.1242/jcs.254276 (PMC8182408; doi:10.1242/jcs.254276)
Supplement: Supplementary information [file joces-134-254276-s1.pdf]

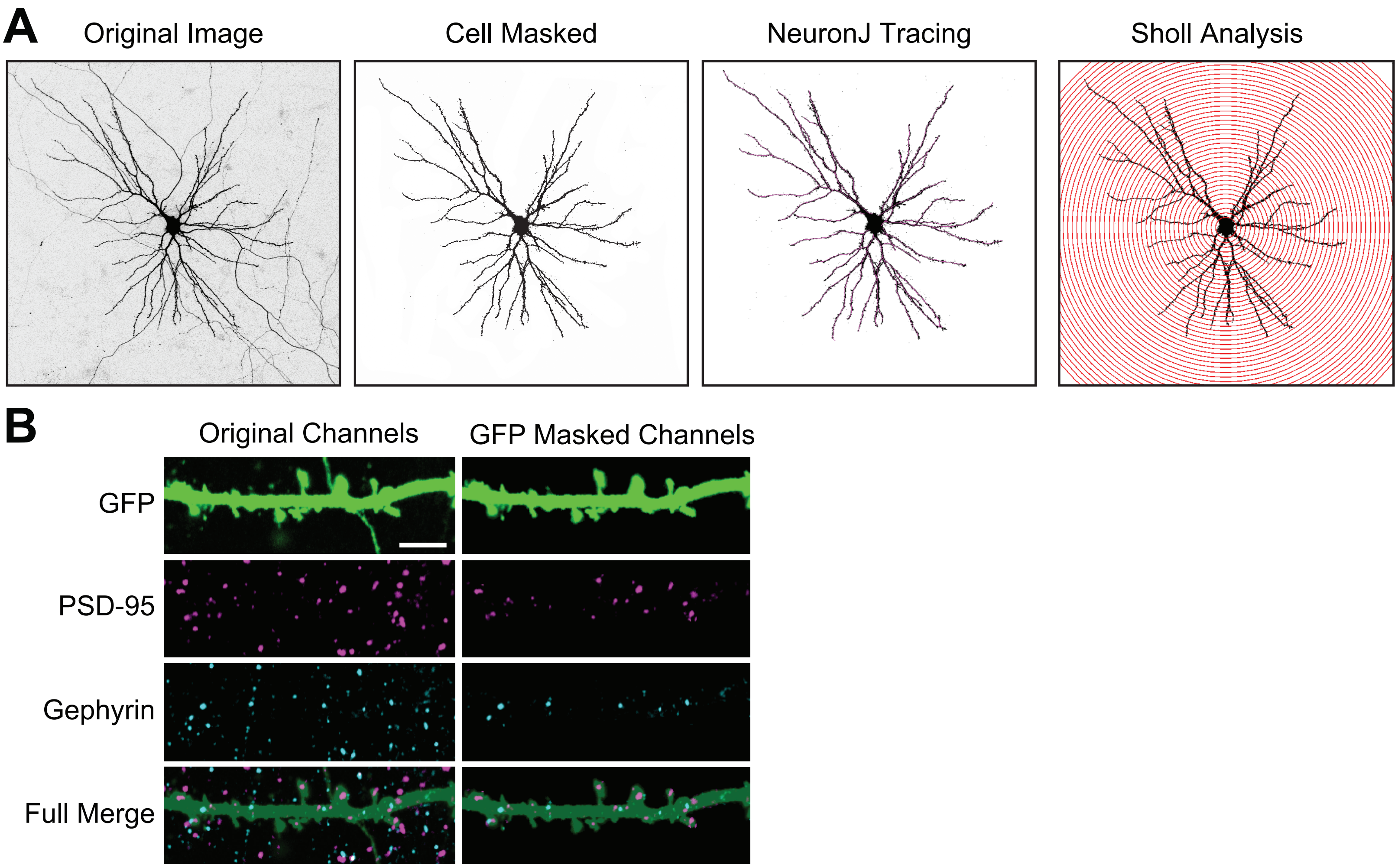

Figure S1 – GFP masking for neurons tracing and quantification of synapse density
(A) Representative eGFP transfected neuron. The original image is manually masked to remove background and axons and then traced using the NeuronJ plugin for ImageJ. (B) Representative eGFP transfected neurons immunostained with the excitatory marker, PSD-95, and the inhibitory marker, gephyrin. The left column shows unmasked images, while the right column is masked using the eGFP channel.

Journal of Cell Science • Supplementary information

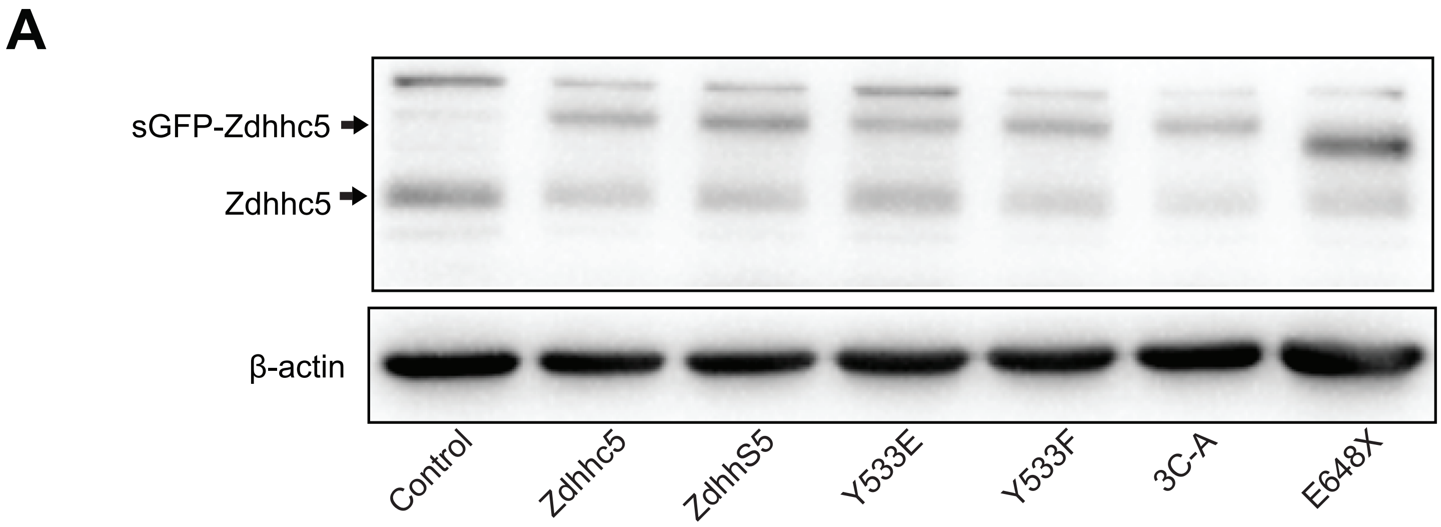

Figure S2 – Expression of Zdhhc5 mutant constructs in primary hippocampal cultures
Hippocampal neurons were nucleofected at 0 DIV with sGFP-tagged Zdhhc5 mutant constructs and lysed at 5 DIV. There is a similar expression of all sGFP-tagged Zdhhc5 variants.
