# Supplementary material for: Regulation of hippocampal excitatory synapses by the Zdhhc5 palmitoyl acyltransferase
Source: J Cell Sci. 2021 May 11;134(9):jcs254276. doi: 10.1242/jcs.254276 (PMC8182408; doi:10.1242/jcs.254276)
Supplement: Reviewer comments [file joces-134-254276_review_history.pdf]

Regulation of hippocampal excitatory synapses by the Zdhhc5 palmitoyl acyltransferase

Jordan J. Shimell, Andrea Globa, Marja D. Sepers, Angela R. Wild, Nusrat Matin, Lynn A. Raymond and Shernaz X. Bamji
DOI: 10.1242/jcs.254276

Editor: Giampietro Schiavo

Review timeline

Original submission:	14 September 2020
Editorial decision:	26 October 2020
First revision received:	22 February 2021
Accepted:	12 March 2021

Original submission

First decision letter

MS ID#: JOCES/2020/254276

MS TITLE: Regulation of Hippocampal Excitatory Synapses by the Zdhhc5 Palmitoyl Acyl Transferase

AUTHORS: Jordan J. Shimell, Andrea K. Globa, Marja D. Sepers, Angela R. Wild, Nusrat Matin, Lynn A. Raymond, and Shernaz X. Bamji
ARTICLE TYPE: Short Report

We have now reached a decision on the above manuscript.

To see the reviewers' reports and a copy of this decision letter, please go to: https://submit-jcs.biologists.org and click on the 'Manuscripts with Decisions' queue in the Author Area. (Corresponding author only has access to reviews.)

As you will see, the reviewers gave favourable reports but raised some critical points that will require amendments to your manuscript. I hope that you will be able to carry these out, because I would like to be able to accept your paper.

We are aware that you may be experiencing disruption to the normal running of your lab that makes experimental revisions challenging. If it would be helpful, we encourage you to contact us to discuss your revision in greater detail. Please send us a point-by-point response indicating where you are able to address concerns raised (either experimentally or by changes to the text) and where you will not be able to do so within the normal timeframe of a revision. We will then provide further guidance. Please also note that we are happy to extend revision timeframes as necessary.

Please ensure that you clearly highlight all changes made in the revised manuscript. Please avoid using 'Tracked changes' in Word files as these are lost in PDF conversion.

I should be grateful if you would also provide a point-by-point response detailing how you have dealt with the points raised by the reviewers in the 'Response to Reviewers' box. Please attend to all of the reviewers' comments. If you do not agree with any of their criticisms or suggestions please explain clearly why this is so.

© 2021. Published by The Company of Biologists under the terms of the Creative Commons Attribution License (https://creativecommons.org/licenses/by/4.0/).

Reviewer 1

Advance summary and potential significance to field

The manuscript by Shimell et al presents new data on the importance of the acyltransferase zDHHC5 in synaptic development/function. The work builds on previous studies by the same group that have shown that the dynamic trafficking of this enzyme regulates access to substrates such as delta catenin and is mediated by phosphorylation-regulated endocytic signals in the large C-terminus. The main conclusions are that zDHHC5 is important for formation of excitatory but not inhibitory synapses, spine density and morphology, and synaptic activity. zDHHC5 is linked to the stability/maturation of silent synapses by the increased ratio of AMPAR relative to NMDAR currents observed in knockout cells. The authors further show the importance of the catalytic activity and localisation of zDHHC5 for these different parameters.
Overall, the work is well performed, appropriately analysed and convincing and makes an important contribution to our wider understanding of the function of the zDHHC family of enzymes in neuronal physiology.

Comments for the author

The authors show that the different zDHHC5 mutants are equally expressed in HEK293 cells (Figure 3A). It would be useful to show similar data in neurons to exclude any neuronal-specific effects e.g. on protein degradation rates.

I didn't fully understand why the Y533F mutant would be decreased at the plasma membrane (Figure 3B). Although this mutation removes the phospho-regulated tyrosine, presumably this Y to F substitution also destroys the endocytic motif. The authors should add some further discussion around this point.

I also didn't fully understand the explanation given for the results of the FRAP experiment (Figure 3E). If I understand correctly, zDHHC5 WT is localised to the plasma membrane and endosomes. I understand why the 3C-A and Y533E mutants, which enhance plasma membrane localisation, lead to reduced mobility but wasn't clear why the Y533F also reduces mobility (albeit by a lesser extent). It would be good to include some additional clarification/discussion of this point.

Reviewer 2

Advance summary and potential significance to field

Shimell et al. here showed that zDHHC5 is a regulatory component for formation and maintenance of excitatory but not inhibitory synapses. They also found that this regulatory role of zDHHC5 in excitatory synapses depends on zDHHC5Â's enzymatic activity, plasma membrane localization and C terminal domain. Most of the key observations are made in cultured rat hippocampal neurons. Key experiments are replicated in zDHHC5 gene trapped animals. Curiously although zDHHC5 GT animals exhibit the same reduction in excitatory synapses as were observed in acute shRNA experiments in rat neurons, this does not translate into a difference in frequency and amplitude of sEPSCs.

Comments for the author

Overall, this study is well-thought and well-executed. I have no methodological concerns. There are some minor omissions that could be improved and clarified in a revision.

Major:
In order to account for the apparently normal excitatory activity in the zDHHC5 GT animals, the authors speculate that there is a selective decrease in the number of silent synapses. Can this be investigated experimentally? For example, PSD95 itself is palmitoylated. Palmitoylation puts PSD95 through certain conformational changes (from a compact to extended configuration) and alters its clustering. Moreover PSD95 is associated with AMPA and NMDA receptor when it is palmitoylated in

© 2021. Published by The Company of Biologists under the terms of the Creative Commons Attribution License (https://creativecommons.org/licenses/by/4.0/).

its extended configuration (Jeyifous et al., 2016 PNAS https://doi.org/10.1073/pnas.1612963113). Have authors checked whether palmitoylation of PSD95 is reduced in their zDHHC5-GT mice? What about NMDA / AMPA receptor densities/localisation? This could help with interpreting their ephys data for AMPA/NMDA current measurement.

Minor:
1. There is a lack of consistency in the nomenclature of the protein of interest. Sometimes ZDHHC5, sometimes zDHHC5 and sometimes Zdhhc5. Suggest you pick one and stick with it.
2. Fig 1D & 1E & 1I: does indicate a significant difference between the zDHHC5 shRNA group and the control shRNA group? What about the comparison between the zDHHC5 shRNA and zDHHC5-R group? This figure might be clearer if you moved the legend out of the way.
3. Lines 107 & 108. Another post-translational modification of zDHHC5 recently described is GlcNAcylation, see PMID 32737405. This regulates substrate recruitment and merits inclusion alongside palmitoylation and phosphorylation.
4. Fig 2B: it isn't clear which group the statistical comparisons are being made with. I assume to control shRNA? Is it not also meaningful to present a comparison of the various zDHHC5-R constructs to the zDHHC5-shRNA group to indicate which of the 'R' constructs are changing puncta density and which are not? It may be clearer to present the gephyrin data separately – maybe in the data supplement?
5. Line 124: it is more conventional to describe premature stop codon mutations as 'X' – i.e. E648X
6. Line 139: the numbering of the zDHHC5 C tail palmitoylation sites is incorrect. This should be cys 236, cys 237, cys 245. Palmitoylation at these sites has been hypothesised (PMID: 19801377) and recently established (PMID 32737405) to control substrate recruitment. Please check numbering of these sites throughout and confirm that the mutagenesis strategy to make these mutants (line 227 onwards) is correctly described. The findings by others that palmitoylation of these sites changes zDHHC5 interaction with some substrates merits discussion. It appears not to modify substrate recruitment in your study, correct? But could a reduced ability to recruit substrates explain why enhanced surface localisation of this mutant does not enhance synapse formation?
7. Line 153: given the deletion of the PDZ binding motif in zDHHC5-E648X, is it surprising that the mobility of this protein in the synapse is unchanged?
8. Line 176: the significance of reduced capacitance may not be immediately obvious to some readers. Suggest you could explain this measures surface area of the cell? I don't understand the significance of the resistance (4F) and holding current (4G) being unchanged.
9. The mouse model is not described in the methods. Given the importance of the mouse model to the study some description is necessary – regardless of the fact that it has been published before. Is this is a knockout? If not, what causes the absence of zDHHC5? Are experimental comparisons in Fig 4 made to littermates?
10. Information about ethical review of animal experiments is missing from the methods.
11. Supplemental Figure 1(B) – the legend says the right column shows unmasked images, but the figure says this column is masked. Which is correct?
12. A recent study about zDHHC5 trafficking and its control by phosphorylation of tyrosine 91 appears to be relevant and should be discussed. PMID: 32958780

Reviewer 3

Advance summary and potential significance to field

The authors have investigated the function of ZDHHC5 in synapse formation/maintenance and have determined that DHHC5 is essential for the formation of excitatory but not inhibitory neurons. This requirement for DHHC5 is dependent on its catalytic activity, indicating that palmitoylation of specific substrates is involved. Analysis of other DHHC5 mutants also revealed that the localisation of DHHC5 at the plasma membrane is also important for its regulation of excitatory synapse formation. The authors went on to provide convincing validation of these findings in vivo by demonstrating a selective decrease in the density of excitatory synapses in DHHC5-GT mice using electron microscopy as well as finding a significantly higher AMPAR/NMDAR ratio in DHHC5-GT mice which may indicate a loss of silent synapses.

© 2021. Published by The Company of Biologists under the terms of the Creative Commons Attribution License (https://creativecommons.org/licenses/by/4.0/).

This is an important study which further demonstrates a clear role for DHHC5 in synapse formation and function. I want to recommend that it is accepted subject to the comments below being addressed.

Comments for the author

The R182A mutant is referred to as a biotinylation control; the authors might want to explain that this is the only extracellular site of biotinylation in the results/methods.

Given that one of the major conclusions of this study is that DHHC5 selectively promotes excitatory synapse formation, it is important to comment about the expression of DHHC5 at excitatory versus inhibitory synapses. From the authors' previous work (PMID: 26334723), they found that 57% of DHHC5 co-localises with PSD-95 whilst 31% co-localises with gephyrin.

Given that DHHC5 is present in both inhibitory and excitatory synapses, then this would suggest a different mechanism potentially through the differential expression of DHHC5 substrates in these different synapse types. Is the effect simply mediated by the presence of PSD-95 in excitatory synapses, to which DHHC5 binds?

In Figure 2B, it might make comparisons of mutants with controls easier if the PSD-95 data and the gephyrin data were separated into two separate bar charts.

In the Li at al 2010 paper describing the DHHC5 GT mouse line, expression of DHHC5 was estimated to be about 7% of WT. Do the authors not detect this residual expression?

There are aspects of this paper which warrant more discussion, for example, is the decrease in the density of PSD-95 positive synapses in the DHHC5_GT mice due to a reduction in the formation and/or the stability of these synapses? The reduction in the mobility of the 3C-A mutant is striking, but the potential mechanism underlying this observation is not discussed.

First revision

<u>Author response to reviewers' comments</u>

We thank all reviewers for their thorough reviews, supportive comments and excellent suggestions. We include point-by-point responses below:

Reviewer 1
The authors show that the different zDHHC5 mutants are equally expressed in HEK293 cells (Figure 3A). It would be useful to show similar data in neurons to exclude any neuronal-specific effects e.g. on protein degradation rates.

We agree with the reviewer and have now tested the expression of the variants in neurons to account for potential neuronal-specific effects on protein degradation rates etc. We now show that all sGFP-tagged Zdhhc5 constructs express similarly in neurons (Suppl Fig 2).

I didn't fully understand why the Y533F mutant would be decreased at the plasma membrane (Figure 3B). Although this mutation removes the phospho-regulated tyrosine, presumably this Y to F substitution also destroys the endocytic motif. The authors should add some further discussion around this point.

The reviewer is correct that Y to F mutations in the tyrosine-based internalization motifs of some proteins can disrupt binding to the AP-2 endocytic adaptor complex and thereby disrupt endocytosis (e.g. PMID: 19903874; PMID: 8918456; PMID: 8910552; PMID: 9175836). However, Y to F mutations in other proteins have been shown to retain AP2μ binding and internalization, and reduce the surface biotinylation of the protein similar to what we have shown for Zdhhc5. Short

© 2021. Published by The Company of Biologists under the terms of the Creative Commons Attribution License (https://creativecommons.org/licenses/by/4.0/).

excerpts from these studies and the references are listed below:

- **(PMID: 10652362)** “GST-CT-Y1424F was able to bind AP-2 complexes with as much efficiency as wild-type GST-CT, suggesting that the phenylalanine at position 1424 is capable of participating in the endocytosis signal (Fig. 6 B).” Importantly, this is the same residue as observed in Zdhhc5: “In seven of the CFTR sequences, the motif YDSI is conserved, suggesting that this sequence conforms to the paradigm of a Y*XX*Φ motif and may be an internalization signal for CFTR.”
- **(PMID: 22247549)** “We next examined the effects of the DMß YxxΦ motif upon MARCH-induced down-regulation. … Substitution of Tyr^{230} with phenylalanine, a residue that can partially substitute for tyrosine with respect to interaction with the endocytic machinery, significantly restored MARCH8-induced DM down- regulation (Fig. 5*A*, *lane 5*, and supplemental Fig. 2)”
- **(PMID: 2100204)** “We have shown that efficient internalization of the human transferrin receptor is dependent on the presence of an aromatic amino acid on the cytoplasmic domain. The native receptor has a tyrosine at position 20 of the cytoplasmic domain. Replacement of this tyrosine with phenylalanine or tryptophan does not significantly alter the internalization rate constant.

However, substitution with serine, leucine, or cysteine reduces internalization by nearly threefold (Table 1)”

- **(PMID: 12692221)** “The results of these experiments showed that, except for the phenylalanine substitution (Y22F), all the mutants exhibit levels of synthesis, processing, and transport to the plasma membrane equivalent to the wild-type levels. Interestingly, while the Y22F mutant Env precursor was expressed at a level comparable to that of the wild type and could be detected on the cell surface in a steady-state immunofluorescent staining assay, ***the level of Y22F mutant Env proteins was dramatically reduced in the surface biotinylation assay. It is possible that the Y22F mutation creates a strong endocytosis signal that results in rapid trafficking from the surface***.”

I also didn’t fully understand the explanation given for the results of the FRAP experiment (Figure 3E). If I understand correctly, zDHHC5 WT is localised to the plasma membrane and endosomes. I understand why the 3C-A and Y533E mutants, which enhance plasma membrane localisation, lead to reduced mobility but wasn’t clear why the Y533F also reduces mobility (albeit by a lesser extent). It would be good to include some additional clarification/discussion of this point.

We agree with the reviewer that this data may at first appear somewhat confounding. While we do not have a definitive explanation at this time, as per the reviewer’s request, we discuss one plausible explanation.
The amount of protein at the cell surface depends on how quickly it is turned over through the endocytic cycle. However, the mobility of a protein within a spine depends on both the rate of endocytosis as well as lateral diffusion along the membrane. We submit that less Y533F at the surface and more stability at spines could be accounted for by both an increase in Y533F in the recycling endosome pool and a decrease in lateral mobility of Y533F along the membrane. It is at present unclear how much of a contribution each of these delivery mechanisms contributes to the overall recovery in our Zdhhc5 FRAP curves.
Further delineation of these mechanisms to fully interpret our data would require us to study the two populations in isolation. This is now discussed in Lines 160-166.
Studying lateral diffusion of proteins in and out of synapses has previously been done using quantum dot analysis (PMID:17481397, PMID 19607795, PMID 18003820, PMID 29790493). Alternatively, it may be possible to generate constructs for our Zdhhc5 mutants with an extracellular loop SEP tag to selectively study the surface fraction of Zdhhc5 mutants. Because of the time frame to set up these more complex experiments in our lab, we submit it is beyond the scope of the current manuscript.

© 2021. Published by The Company of Biologists under the terms of the Creative Commons Attribution License (https://creativecommons.org/licenses/by/4.0/).

Reviewer 2

Overall, this study is well-thought and well-executed. I have no methodological concerns. There are some minor omissions that could be improved and clarified in a revision.

Major:

In order to account for the apparently normal excitatory activity in the zDHHC5 GT animals, the authors speculate that there is a selective decrease in the number of silent synapses. Can this be investigated experimentally? For example, PSD95 itself is palmitoylated. Palmitoylation puts PSD95 through certain conformational changes (from a compact to extended configuration) and alters its clustering. Moreover, PSD95 is associated with AMPA and NMDA receptor when it is palmitoylated in its extended configuration (Jeyifous et al., 2016 PNAS https://doi.org/10.1073/pnas.1612963113). Have authors checked whether palmitoylation of PSD95 is reduced in their zDHHC5-GT mice? What about NMDA / AMPA receptor densities/localisation? This could help with interpreting their ephys data for AMPA/NMDA current measurement.

The reviewer brings up an interesting theory. Jeyifous et al., 2016 (PMID:27956638) do show that PSD95 is associated with AMPA and NMDA receptors when it is palmitoylated in its extended configuration. Indeed, alterations in PSD95 palmitoylation are linked to changes in the AMPAR content of the PSD, but these changes occur in the same direction. That is to say that if PSD95 palmitoylation is reduced, that would be associated with a reduction in AMPAR expression in the PSD and vice-versa.

Importantly, the same paper shows that manipulations that alter PSD95 palmitoylation in the PSD have no effect on NMDAR expression in the synapse and the paper concludes that general inhibitors/enhancers of PAT activity (such as 2-BP or palmostatin) are probably working through PSD-localized DHHC2 (in the case of PSD95). So, if alterations in the PAT activity of Zdhhc5 were to impact the palmitoylation of PSD95 (eg to decrease it) and this was believed to explain the reduction in PSD95 punctae, this would further be expected to reduce AMPAR expression at the synapse while having no effect on NMDAR composition, reducing the AMPAR:NMDAR ratio. As we have observed the opposite in the GeneTrap Zdhhc5 mice (Figure 4M; an increase in the AMPAR:NMDAR ratio) we believe that the findings of this paper cannot explain our results. We therefore return to our initial interpretation that there is a selective decrease in the number of silent synapses.

Minor:

1. There is a lack of consistency in the nomenclature of the protein of interest. Sometimes ZDHHC5, sometimes zDHHC5 and sometimes Zdhhc5. Suggest you pick one and stick with it.

We apologize for the confusion. As we have stated in the paper, we are using ZDHHC5 as the preferred HUGO Human nomenclature (lines 41/42) and Zdhhc5 as the preferred

HUGO mouse/rodent nomenclature (lines 68/69). We have corrected the instances of zDHHC5 and we thank the reviewer for their attention to detail.

*2. Fig 1D & 1E & 1I: does *** indicate a significant difference between the zDHHC5 shRNA group and the control shRNA group? What about the comparison between the zDHHC5 shRNA and zDHHC5-R group? This figure might be clearer if you moved the legend out of the way.*

We apologize if this figure was not initially clear. We have now; 1) added lines above the bars demonstrating which groups are being compared, 2) compared zDHHC5 shRNA and zDHHC5-R groups, and 3) moved the legends to hopefully enhance the clarity of this figure.

3. Lines 107 & 108. Another post-translational modification of zDHHC5 recently described is GlcNAcylation, see PMID 32737405. This regulates substrate recruitment and merits inclusion alongside palmitoylation and phosphorylation.

We thank the reviewer for pointing out this oversight. We agree that this paper merits inclusion in our discussion alongside palmitoylation and phosphorylation and have included this reference (now in lines 106/107).

4. Fig 2B: it isn’t clear which group the statistical comparisons are being made with. I assume

© 2021. Published by The Company of Biologists under the terms of the Creative Commons Attribution License (https://creativecommons.org/licenses/by/4.0/).

to control shRNA? Is it not also meaningful to present a comparison of the various zDHHC5-R constructs to the zDHHC5-shRNA group to indicate which of the 'R' constructs are changing puncta density and which are not? It may be clearer to present the gephyrin data separately – maybe in the data supplement?

We agree with the reviewer that a comparison with shRNA condition in addition to control would be informative. In addition to asterisks to denote significant differences to control, we have added hashtags to denote significant differences to shRNA. We respectfully believe that showing the gephyrin data alongside the PSD-95 data reflects an additional control for cell heath, culture density and other factors that may impact synapse density.

5. Line 124: it is more conventional to describe premature stop codon mutations as 'X' – i.e. E648X

We agree with the reviewer and have now changed this nomenclature throughout.

6. Line 139: the numbering of the zDHHC5 C tail palmitoylation sites is incorrect. This should be cys 236, cys 237, cys 245. Palmitoylation at these sites has been hypothesised (PMID: 19801377) and recently established (PMID 32737405) to control substrate recruitment. Please check numbering of these sites throughout and confirm that the mutagenesis strategy to make these mutants (line 227 onwards) is correctly described. The findings by others that palmitoylation of these sites changes zDHHC5 interaction with some substrates merits discussion. It appears not to modify substrate recruitment in your study, correct? But could a reduced ability to recruit substrates explain why enhanced surface localisation of this mutant does not enhance synapse formation?

We apologize for the error in numbering, and have now indicated the correct cysteine residues (236, 237, 245) as per the reviewer. The mutagenesis strategy described is correct for these residues; this was a plain error and we are very grateful to the reviewer for pointing this out.

We also agree with the reviewer that altering Zdhhc5 may change substrate interaction, and have accordingly included a sentence in the manuscript. Line 155-158: "This may be due to alterations in substrate interaction and/or recruitment, as the mutants that display the greatest surface localization have alterations in amino acid residues known to be important in these processes (Brigidi et al., 2015; Woodley & Collins, 2019)".

7. Line 153: given the deletion of the PDZ binding motif in zDHHC5-E648X, is it surprising that the mobility of this protein in the synapse is unchanged?

Our previous paper (PMID: 26334723) demonstrated that deletion of the PDZ binding motif did not affect basal protein mobility, and so we did not have reason to believe that the E648X mutation (which deletes the PDZ domain) would affect protein mobility.

8. Line 176: the significance of reduced capacitance may not be immediately obvious to some readers. Suggest you could explain this measures surface area of the cell? I don't understand the significance of the resistance (4F) and holding current (4G) being unchanged.

We agree that the significance of the reduced capacitance may not be obvious to some. We have therefore added in lines starting at line 184: "*As Zdhhc5 did not impact dendritic length or complexity in vitro (Figure 1A, B), and Zdhhc5-GT mice have a lower density of excitatory synapses (Figure 4C), this reduced capacitance may be due to fewer spines resulting in less total membrane. The lack of change in the resistance (4F) and holding current (4G) when voltage-clamped at -70 mV suggests that the composition of the channels open in the cells at this potential is unchanged and the cells do not have altered excitability*".

The lack of change in membrane resistance and holding current suggests that the composition of channels (mostly K channels) open in the cells at -70mV is unchanged and that the cells have not become more or less leaky/excitable. However, we cannot make claims on the excitability of the cells without further testing of action potentials in response to current steps – while we did measure voltage changes in response to current steps we found no difference in the IV curve or

© 2021. Published by The Company of Biologists under the terms of the Creative Commons Attribution License (https://creativecommons.org/licenses/by/4.0/).

minimum current required to elicit action potentials, suggesting no differences in excitability between genotypes. We are happy to include this negative data if you feel it will enhance the manuscript.

9. The mouse model is not described in the methods. Given the importance of the mouse model to the study some description is necessary – regardless of the fact that it has been published before. Is this is a knockout? If not, what causes the absence of zDHHC5? Are experimental comparisons in Fig 4 made to littermates?

We have now added a section to the Methods, starting at line 220, briefly describing the Zdhhc5 GeneTrap mice as follows: *"Zdhhc5-GT mice were obtained from the Don Hilgemann (UT Southwestern). The mice were originally generated by Li et al., (2010) from an embryonic stem cell line (RRD533, strain 129/Ola) with an insertional mutation in Zdhhc5 from the International Gene Trap Consortium (Skarnes et al., 2004; Nord et al., 2006). A gene-trapping vector, pGT11xf, introduced an in-frame fusion between the 5' exons of the trapped gene and a reporter.*

All experimental comparisons from Fig 4 are made with WT age-matched littermate controls. We have amended the Fig 4 Legend to include this with a statement at the end: *"All data is from comparisons of Zdhhc5-GT with age-matched littermate controls and is shown as mean ± SEM".*

10. Information about ethical review of animal experiments is missing from the methods.

We apologize for this oversight. We have added a section to the Methods, starting at line 218, as follows:
"All experimental procedures and housing conditions were approved by the UBC Animal Care Committee and were in accordance with the Canadian Council on Animal Care (CCAC) guidelines".

11. Supplemental Figure 1(B) - the legend says the right column shows unmasked images, but the figure says this column is masked. Which is correct?

We thank the reviewer for bringing this to our attention, and have correctly labelled the figure as the left showing unmasked images while the right is showing the masked images.

12. A recent study about zDHHC5 trafficking and its control by phosphorylation of tyrosine 91 appears to be relevant and should be discussed. PMID: 32958780

We agree that this is an important paper for understanding the mechanism of Zdhhc5 function. We have added a brief discussion of this paper to lines 139-142 as follows: "Recent work by Hao and colleagues have also shown that phosphorylation of tyrosine residue Y91 near the DHHC domain can decrease ZDHHC5 activity, further underscoring the role of tyrosine phosphorylation in ZDHHC5 function (Hao et al., 2020)."

Reviewer 3
The R182A mutant is referred to as a biotinylation control; the authors might want to explain that this is the only extracellular site of biotinylation in the results/methods.

We have now added this information to our figure legends where it will be easily accessible for readers and we thank the reviewer for their suggestion.

Given that one of the major conclusions of this study is that DHHC5 selectively promotes excitatory synapse formation, it is important to comment about the expression of DHHC5 at excitatory versus inhibitory synapses. From the authors' previous work (PMID: 26334723), they found that 57% of DHHC5 co-localises with PSD-95 whilst 31% co-localises with gephyrin.

We thank the reviewer for their suggestion, and have added this into our results and discussion around Figure 1 (lines 88-91): *"This is consistent with our previous finding that Zdhhc5 is*

© 2021. Published by The Company of Biologists under the terms of the Creative Commons Attribution License (https://creativecommons.org/licenses/by/4.0/).

primarily localized to excitatory synapses (57% of Zdhhc5 colocalizes with PSD-95, while 31% co-localizes with gephyrin (Brigidi et al., 2015)).”

Given that DHHC5 is present in both inhibitory and excitatory synapses, then this would suggest a different mechanism potentially through the differential expression of DHHC5 substrates in these different synapse types. Is the effect simply mediated by the presence of PSD-95 in excitatory synapses, to which DHHC5 binds?

Localization of PATs is critical for their function, and in the case of Zdhhc5 seems to be particularly relevant due to its dynamic and mobile nature. We would agree with the reviewer that Zdhhc5 probably localizes to excitatory and inhibitory synapses through different mechanisms and/or binding partners. Indeed, some of the other studied Zdhhc5 substrates, including Grip1 and Furin have been suggested to have roles at inhibitory synapses (Grip1 PMID: 15451408; Furin PMID: 30333479). We do not believe that the effect is mediated simply by the presence of PSD-95 in excitatory synapses, based on the other papers that demonstrate that the C-tail palmitoylation affects substrate interactions (PMID: 31402609), and/or the requirement of Fyn and/or LYN kinases for specific substrate interactions (PMID: 26334723; PMID: 32958780).

In Figure 2B, it might make comparisons of mutants with controls easier if the PSD-95 data and the gephyrin data were separated into two separate bar charts.

We would be happy to move the data into two separate graphs if that is the editorial decision. We prefer showing the data in one graph as PSD95 and gephyrin density were collected in the same cells and provides an extra internal control for cell heath, culture density and other factors that may impact synapse density.

In the Li at al 2010 paper describing the DHHC5 GT mouse line, expression of DHHC5 was estimated to be about 7% of WT. Do the authors not detect this residual expression?

We appreciate the reviewer’s knowledge of this field. The Li et al (2010) paper states that, “*the amount of DHHC5 protein produced in numerous experiments was determined to be **no greater** than 7% in brain tissue, as estimated by immunoblotting*.” Similar to the Li et al (2010) paper, at normal exposure levels we did not detect any bands indicating residual expression (which they only saw at a 5 minute exposure with over saturated bands, which we did not do).

There are aspects of this paper which warrant more discussion, for example, is the decrease in the density of PSD-95 positive synapses in the DHHC5_GT mice due to a reduction in the formation and/or the stability of these synapses? The reduction in the mobility of the 3C-A mutant is striking, but the potential mechanism underlying this observation is not discussed.

Unfortunately, discussion of this would amount to speculation without additional experiments. We do see reduced excitatory synapses in the Zdhhc5GT, but cannot be sure if this is due to a reduction in formation, maintenance, or stability of these synapses. The reduction of mobility in the 3CA mutant is discussed a length in the Woodley & Collins, 2019 paper where they suggest that the C-terminal mutant is unable to be internalized because it is not palmitoylated on the C-terminus, which raised the possibility that interaction with Golga7b functions in some protective manner, preventing the endocytosis of Zdhhc5.

Second decision letter

MS ID#: JOCES/2020/254276

MS TITLE: Regulation of Hippocampal Excitatory Synapses by the Zdhhc5 Palmitoyl Acyl Transferase

AUTHORS: Jordan J. Shimell, Andrea K. Globa, Marja D. Sepers, Angela R. Wild, Nusrat Matin, Lynn A. Raymond, and Shernaz X. Bamji
ARTICLE TYPE: Short Report

© 2021. Published by The Company of Biologists under the terms of the Creative Commons Attribution License (https://creativecommons.org/licenses/by/4.0/).

I am delighted to tell you that your manuscript has been accepted for publication in Journal of Cell Science, pending standard ethics checks.

Reviewer 1

Advance summary and potential significance to field

.

Comments for the author

Thank you for addressing my comments. I have no further questions and am happy with the submitted manuscript.

Reviewer 2

Advance summary and potential significance to field

Shimell et al. show that Zdhhc5 is a regulatory component for formation and maintenance of excitatory but not inhibitory synapses. They report that this regulatory role of Zdhhc5 in excitatory synapses depends on Zdhhc5Â’s enzymatic activity, plasma membrane localization and C terminal domain. This opens a new regulatory role for this particular enzyme. The same research group previously demonstrated that Zdhhc5 regulates plasticity of synaptic connections. The significance to the field of these new findings is that the presence, subcellular location, post-translational modifications and activity of Zdhhc5 are all found to be required for formation of excitatory synapses during development. Defects in Zdhhc5 signaling may therefore contribute to a number of neuropathologies.

Comments for the author

No further comments. I thank the authors for their attention to detail in the revision.

Reviewer 3

Advance summary and potential significance to field

The authors have investigated the function of ZDHHC5 in synapse formation/maintenance and have determined that DHHC5 is essential for the formation of excitatory but not inhibitory neurons. This requirement for DHHC5 is dependent on its catalytic activity, indicating that palmitoylation of specific substrates is involved. Analysis of other DHHC5 mutants also revealed that the localisation of DHHC5 at the plasma membrane is also important for its regulation of excitatory synapse formation. The authors went on to provide convincing validation of these findings in vivo by demonstrating a selective decrease in the density of excitatory synapses in DHHC5-GT mice using electron microscopy as well as finding a significantly higher AMPAR/NMDAR ratio in DHHC5-GT mice which may indicate a loss of silent synapses.

Comments for the author

I am satisfied that authors have addressed my points in this revised version of the manuscript.

© 2021. Published by The Company of Biologists under the terms of the Creative Commons Attribution License (https://creativecommons.org/licenses/by/4.0/).
